# Atypical Phenotype in a Spinocerebellar Ataxia Type 2 Kindred

**DOI:** 10.5334/tohm.639

**Published:** 2021-08-04

**Authors:** Shweta Prasad, Vikram V. Holla, Pramod Kumar Pal

**Affiliations:** 1 Department of Clinical Neurosciences, National Institute of Mental Health & Neurosciences, Hosur Road, Bengaluru-560029, Karnataka, India; 2 Department of Neurology, National Institute of Mental Health & Neurosciences, Hosur Road, Bengaluru-560029, Karnataka, India

**Keywords:** SCA2, perioral twitches, dystonia, SCA3

## Abstract

**Background:**

Non-ataxic manifestations in autosomal dominant cerebellar ataxias are variable and influenced by CAG repeat length and age at onset. This report describes a genetically proven SCA2 kindred with an atypical phenotype resembling SCA3.

**Case Report:**

The phenotype of five genetically proven patients with SCA2 in this report differed from the typical phenotype owing to persistence of reflexes late into the course of illness, absence of peripheral neuropathy, and very prominent facial twitches.

**Discussion:**

Despite descriptions of typical phenotypes of SCA, significant variations occur, especially within kindreds. Caution should be exercised in clinical diagnoses of SCA, especially with atypical features.

## Introduction

Autosomal dominant cerebellar ataxias (ADCA) are a group of clinically and genetically heterogenous disorders characterized by progressive cerebellar ataxia associated with a multitude of varying neurological and systemic features [1]. Although genetic testing is the mainstay for diagnosing these disorders, several ADCAs have specific combinations of signs which may suggest a specific variant. For instance, spinocerebellar ataxia type 2 (SCA2) is an ADCA typically characterized by ataxia associated with early saccadic slowing, hyporeflexia, tremor and myoclonus [2]. On the contrary, spinocerebellar ataxia type 3 (SCA3), is characterized by ataxia associated with pyramidal signs, dystonic-rigid extrapyramidal syndrome, hyperreflexia, peripheral amyotrophy, facial atrophy, facial and lingual and fasciculations [3]. The non-ataxic manifestations may be variable, and are highly influenced by the CAG repeat length and age at onset [4]. This report describes a genetically proven SCA2 kindred with an atypical phenotype resembling SCA3.

**Figure 1:**
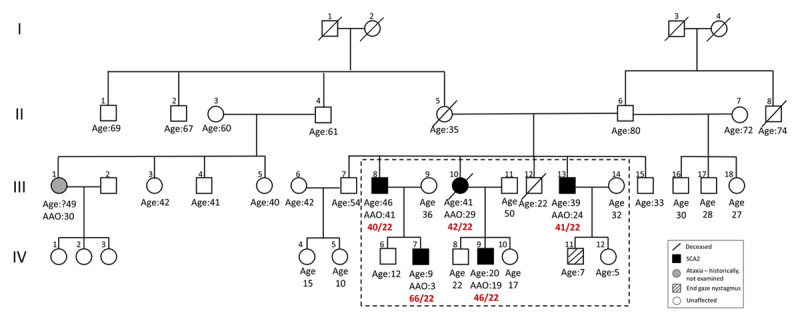
Pedigree chart.

A retrospective chart review was performed for five genetically proven patients with SCA2 from a single family (***Figure 1***) who presented to the Neurology outpatient department. Videos of the patients were taken after written informed consent.

## Case series

### Case 1 (III:13)

The proband presented at the age of 37, with a 13-year history of imbalance while walking, 11-year history of slurring of speech, head tremors for 10 years, and upper limb (UL) tremors for 2–3 years. On examination, apart from definite cerebellar signs, he had supranuclear upgaze restriction, and slow saccades with head thrust (***Video 1***). On opening his mouth, he developed perioral twitching predominantly involving the ride side of the mouth. He had parkinsonism, in addition to dystonia involving the neck, left UL, and tremor of head and UL. A combination of spasticity and rigidity was observed in both lower limbs (LL). UL deep tendon reflexes (DTR) were normal, whereas LL DTRs were exaggerated with ankle clonus. Nerve conduction studies (NCS), electromyogram (EMG), visual evoked potentials (VEP), and somatosensory evoked potentials (SSEP) were normal (***Table 1***). Brainstem auditory evoked response (BAER) was abnormal bilaterally, and magnetic resonance imaging (MRI) brain revealed pontocerebellar atrophy. His CAG repeat length was 41/22. At a 2-year follow up (***Video 2***), the patient was wheelchair bound with worsening of all features. There was significant temporal hollowing, with an increase in perioral twitching, tremor, parkinsonism. LL DTRs persisted to be exaggerated with ankle clonus.

**Video 1:** Case 1 (III:13). Segment 1: Cerebellar signs, hypomimia, supranuclear upgaze restriction, and slow saccades with head thrust, perioral twitching, dystonia, and brisk DTR.

**Table 1:** Clinical characteristics and investigations.


	CASE 1 (III:13)	CASE 2 (III:10)	CASE 3 (III:8)	CASE 4 (IV:7)	CASE 5 (IV:9)

Gender	M	F	M	M	M

Age* (years)	37	40	45	9	20

AAO (years)	24	29	41	3	19

DOI(years)	13	11	4	6	1

CAG	41/22	42/22	40/22	66/22	46/22

EOM					

–Saccades	Slow	Slow	Slow	Slow	Slow

–Head thrust	+	+	+	+	–

Twitches	Perioral	Perioral, Tongue	Perioral	Perioral	Lumbar paraspinal

Tone					

- LL Spasticity	+	+	+	+	+

- Rigidity	+	+	+	+	–

Tremor	Head, UL	Head, UL	Head, UL Tongue	UL, Tongue	–

Dystonia	Cervical, UL, Trunk	UL	Cervical, UL, Trunk	UL	–

Parkinsonism	+	+	+	+	–

DTR	KJ Brisk	KJ Brisk	Diminished	Diminished	KJ Brisk

NCS	Normal	Normal	Normal	Normal	UL sensory neuropathy

EMG	Normal	Normal	Normal	Normal	Normal^#^

VEP	Normal	Normal	Normal	Prolonged	Normal

BAER	Abnormal bilaterally	Normal	Normal	Normal	Normal

SSEP	Normal	Normal	Normal	Normal	Normal

MRI	PC atrophy	PC atrophy	PC atrophy	PC atrophy	Cerebellar atrophy


* Age at last evaluation; #: Paraspinal EMG not done. AAO: Age at onset; BAER: Brainstem auditory evoked response; DTR: Deep tendon reflex; EMG: Electromyography; EOM: Extraocular movements; F: Female; KJ: Knee jerk; LL: Lower limb; M: Male; MRI: Magnetic resonance imaging; NCS: Nerve conduction studies; PC: Pontocerebellar; SSEP: Somatosensory evoked potentials; UL: Upper limb; VEP: Visual evoked potentials.

**Video 2:** Case 1 (III:13). Segment 2: At 2-year follow-up, significant temporal hollowing, with an increase in perioral twitching, and tremor. LL DTR persisted to be exaggerated with ankle clonus.

### Case 2 (III:10)

Elder sister of the proband, presented at the age of 40 years, with a 11-year history of progressive imbalance while walking, slurring of speech, and twitching of the mouth. Her phenotype was similar to the proband with presence of cerebellar signs, dystonia, and parkinsonism. The main difference was a significantly worse tremor, twitches involving the tongue and dystonia limited to the UL (***Video 3***). All investigations were normal. Her CAG repeat length was 42/22. The patient progressively worsened, and expired at the age of 42 years.

**Video 3:** Case 2 (III:10). Similar to video 1, except for significantly worse tremor, twitches involving the tongue and dystonia limited to the UL.

### Case 3 (III:8)

Elder brother of the proband, presented at the age of 46 years, with imbalance and swaying while walking, slurring of speech, incoordination of UL for 5 years. He also gave history of occasional nasal regurgitation, with sporadic fasciculations, the sites of which were uncertain. His phenotype was also similar to the proband, except that dystonia was significantly worse and predominantly involved the UL and trunk, there was a significant tongue tremor, parkinsonism was worse, and DTRs were diminished (***Video 4***). MRI brain showed pontocerebellar atrophy, and the rest of his investigations were normal. His CAG repeat length was 40/22. The patient was lost to follow up.

**Video 4:** Case 3 (III:8). Similar to video 1, except for dystonia that was significantly worse, and predominantly involved the UL and trunk, with significant tongue tremor.

### Case 4 (IV:7)

Second son of case 3, presented at the age of 7 years, with a 4-year history of reduced facial expression, and drooling of saliva. Slowness of activities and slurred speech for 3 years, and imbalance and swaying while walking for 1 year. On examination (***Video 5***), he had head thrusts, perioral twitches, jerky UL tremor and tongue tremor, UL dystonia, and cerebellar signs. He had LL spasticity and rigidity, and DTR were diminished. His VEP was prolonged, MRI brain showed pontocerebellar atrophy and rest of the investigations were normal. His CAG repeat length was 66/22. At follow-up after 2 years (***Video 6***), there was significant worsening in cerebellar signs, and parkinsonism. He had developed supranuclear gaze palsy and had persistent drooling of saliva.

**Video 5:** Case 4 (IV:7). Segment 1: Head thrusts, perioral twitches, jerky UL tremor and tongue tremor, UL dystonia, cerebellar signs, and DTR were diminished.

**Video 6:** Case 4 (IV:7). Segment 2: At 2-year follow-up. Significant worsening in cerebellar signs., with supranuclear gaze palsy and persistent drooling of saliva.

### Case 5 (IV:9)

Second son of case 2, presented at the age of 19 with a 1-year history of imbalance while walking. On examination, he had mild supranuclear upgaze restriction, and slow saccades. Occasional twitches were observed in the lumbar paraspinal muscles (***Video 7***). He had cerebellar signs, spasticity in LL and brisk LL DTR. There was no dystonia, tremor or parkinsonism. NCS revealed UL sensory neuropathy. Cerebellar atrophy was observed on MRI brain. His CAG repeat length was 46/22.

**Video 7:** Case 5 (IV:9). Mild supranuclear upgaze restriction, and slow saccades. Occasional twitches in the lumbar paraspinal muscles, and cerebellar signs.

## Discussion

The cases described in this report differ from the typical SCA2 phenotype owing to the persistence of reflexes late into the course of illness, absence of peripheral neuropathy, and very prominent facial twitches. These features are typically associated with SCA3, and have been seldom reported in SCA2. Rosa et al, reported the presence of brisk DTR, as a distinctive and main feature in a large SCA2 kindred from Argentina [5]. In the same cohort, in comparison to previous reports, peripheral neuropathy was also observed at a milder severity. The exact aetiology and pathogenesis of facial twitches is uncertain, and this feature has been sporadically reported in SCA2. Varying terminologies, ranging from facial action myoclonus [6], perioral fasciculations [2], and perioral myokymia [7] have been utilised. Although degeneration of brain stem motor nuclei may cause these twitches, there is inadequate evidence to support this speculation. Rossi et al., in a systematic review of clinical features in ADCAs, reported the prevalence of pyramidal signs in 11–36%, and myokymia (site unspecified) in 29% of patients with SCA2 [8]. Although these symptoms are reported in SCA2, they are atypical, and are bound to lead to a clinical conundrum. Another interesting observation was the very early onset of illness at 3 years of age in Case 4 (IV:7) which was associated with a high repeat length. This age of onset of SCA2 is rare and till date very few cases have been reported and CAG repeat expansions can be very high as the expansion length is known to inversely correlate with age at onset. The maximum reported CAG repeat length has been 884/22 in a child with disease onset at the age of 3 months [9].

The cases in this cohort also had features typical of SCA2. For instance, all cases had significantly slow saccades which are known to strongly correlate with polyglutamine expansion, and can be present early in the stages of illness, even prior to the onset of other clinical features of SCA2 [10]. Owing to this, slow saccades are often considered to be highly significant and are a critical clue in the diagnosis of SCA2 [10,11]. Parkinsonism is a relatively common non-ataxic manifestation of SCA2, and patients with SCA2 related parkinsonism have been reported to carry low to intermediate range CAG repeats with CAA, CGG, and CGC interruptions [10,12,13].

Modifier genes are known to modulate the phenotypic manifestations of target genes by producing wide range of effects such as dominance modification, reduced penetrance, expressivity and phenotypic pleiotropy [14]. Although the exact basis for varying phenotypes within a genotype is uncertain, the possibility of a modifier gene should be considered especially in the presence of similar atypical features within a family.

## Conclusion

This report described a SCA2 kindred with atypical features such as persistence of brisk reflexes, prominent perioral twitches, and lack of peripheral neuropathy, all of which are observed in SCA3. Despite the descriptions of typical phenotypes of SCA, significant variations may occur, especially within kindreds. Caution should be exercised in the clinical diagnoses of SCA subtypes, especially when patients have atypical features.
